# Cerebral Infarction in Childhood-Onset Craniopharyngioma Patients: Results of KRANIOPHARYNGEOM 2007

**DOI:** 10.3389/fonc.2021.698150

**Published:** 2021-07-14

**Authors:** Svenja Boekhoff, Brigitte Bison, Daniela Genzel, Maria Eveslage, Anna Otte, Carsten Friedrich, Jörg Flitsch, Hermann L. Müller

**Affiliations:** ^1^ Department of Pediatrics and Pediatric Hematology/Oncology, University Children’s Hospital, Carl von Ossietzky University, Klinikum Oldenburg AöR, Oldenburg, Germany; ^2^ Department of Neuroradiology, University Hospital Würzburg, Würzburg, Germany; ^3^ Institute of Biostatistics and Clinical Research, University of Münster, Münster, Germany; ^4^ Department of Neurosurgery, University Hospital Hamburg-Eppendorf (UKE), Hamburg, Germany

**Keywords:** craniopharyngioma, cerebral infarction, surgery, irradiation, quality of life

## Abstract

**Background:**

Cerebral infarction (CI) is a known vascular complication following treatment of suprasellar tumors. Risk factors for CI, incidence rate, and long-term prognosis are unknown for patients with childhood-onset craniopharyngioma (CP).

**Methods:**

MRI of 244 CP patients, recruited between 2007 and 2019 in KRANIOPHARYNGEOM 2007, were reviewed for CI. Risk factors for CI and outcome after CI were analyzed.

**Results:**

Twenty-eight of 244 patients (11%) presented with CI based on reference assessment of MRI. One CI occurred before initial surgery and one case of CI occurred after release of intracystic pressure by a cyst catheter. 26 of 28 CI were detected after surgical tumor resection at a median postoperative interval of one day (range: 0.5-53 days). Vascular lesions during surgical procedures were documented in 7 cases with CI. No relevant differences with regard to surgical approaches were found. In all 12 irradiated patients, CI occurred before irradiation. Multivariable analyses showed that hydrocephalus and gross-total resection at the time of primary diagnosis/surgery both were risk factors for CI. After CI, quality of life (PEDQOL) and functional capacity (FMH) were impaired.

**Conclusions:**

CI occurs in 11% of surgically-treated CP cases. Degree of resection and increased intracranial pressure are risk factors, which should be considered in the planning of surgical procedures for prevention of CI.

## Introduction

Childhood-onset, adamantinomatous craniopharyngiomas (CP) are rare embryonal malformational tumors, originating in the sellar and parasellar region with WHO grade I malignancy. Long-term prognosis and quality of life (QOL) are frequently impaired due to sequelae caused by the anatomical location of CP close to the pituitary gland, the hypothalamus, and the optic chiasm (1–5). In CP patients with hypothalamic involvement both survival rates and QOL are reduced (6–8). When compared with the general population, CP patients were observed to present with a 3–19 fold higher cardiovascular mortality rate (9–11). An even higher risk was reported for women with CP (12). A 14% rate of cerebrovascular events has been reported by Regine et al. (13), all in patients with CP who received irradiation doses >61 Gy.

The purpose of our study was to determine the incidence of cerebral infarction (CI) in a cohort of 244 German childhood-onset CP patients recruited between 2007 and 2019 with a high degree of completeness in the prospective, randomized trial KRANIOPHARYNGEOM 2007 (German Childhood Craniopharyngioma Registry) (14, 15). Furthermore, we analyzed outcome and risk factors for CI, based on evaluation of clinical and neuroradiological presentation and treatment modalities in these CI patients when compared to CP patients without CI, recruited in the trial KRANIOPHARYNGEOM 2007 during the same time period.

## Patient Cohorts and Methods

Two hundred and eighty-two patients (143 females/139 males) diagnosed with adamantinomatous CP (median age at CP diagnosis: 9.2 years, ranging from 1.3 to 17.9 years) were recruited between 2007 and 2019 in the trial KRANIOPHARYNGEOM 2007 (Clinical Trial No. NCT01272622) (16) and prospectively observed after a median follow-up interval of 4 years, ranging from 0.01 to 13.1 years). Adamantinomatous CP as the histological diagnosis was confirmed by pathological reference assessment in all cases. In KRANIOPHARYNGEOM 2007, the timepoint of irradiation (XRT) after incomplete resection was randomisized in patients with >5 years of age at diagnosis (arm I: immediate XRT after diagnois *versus* arm II: XRT at the time of progression of the residual tumor). A further secondary endpoint and question of the trial KRANIOPHARYNGEOM 2007 was the rate of CI. The following analyses included 244 patients (125 females/119 males) with available MRI at diagnosis and during a follow-up of at least 56 days after surgery.

### Neuroradiological Diagnostics

According to the KRANIOPHARYNGEOM 2007 protocol (17–19), cranial MRIs were performed at the time point of CP diagnosis and prospectively at 3-months intervals during the first year follow-up. Neuroradiologists (B.B: and D.G.) blinded for clinical information assessed presurgical hypothalamic involvement (HI), tumor volume and location of CP, degree of surgical resection, and surgical hypothalamic lesions (HL). HI of CP was categorized into defined degrees: grade 0 of HI: no detectable HI on presoperative MRIs; grade 1 HI: HI of the anterior hypothalamic area not involving mammillary bodies (MB) and hypothalamic structures dorsal of MB; and grade 2 HI: HI of both anterior and posterior hypothalamic structures, i.e. involving anterior hypothalamic area, MB and hypothalamic structures dorsal of MB (17, 18).

Based on postsurgical MRIs, postsurgical HLs were categorized according to the same criteria in 3 grades: grade 0 HL: no detectable HL on postoperative MRIs; grade 1 HL: HL of anterior hypothalamic structures not involving MB, and grade 2 HL: HL involving anterior hypothalamic areas, MB and hypothalamic structures dorsal of MB. The tumor size of CP was calculated using the formula “½ (A x B x C)” (aligned to the ellipsoid model: 4/3 π [A x B x C]), where A, B and C are the maximum dimensions in the standard planes: axial (transverse, A), coronal (craniocaudal, B) and sagittal (anteroposterior, C).

**Figure 1:**
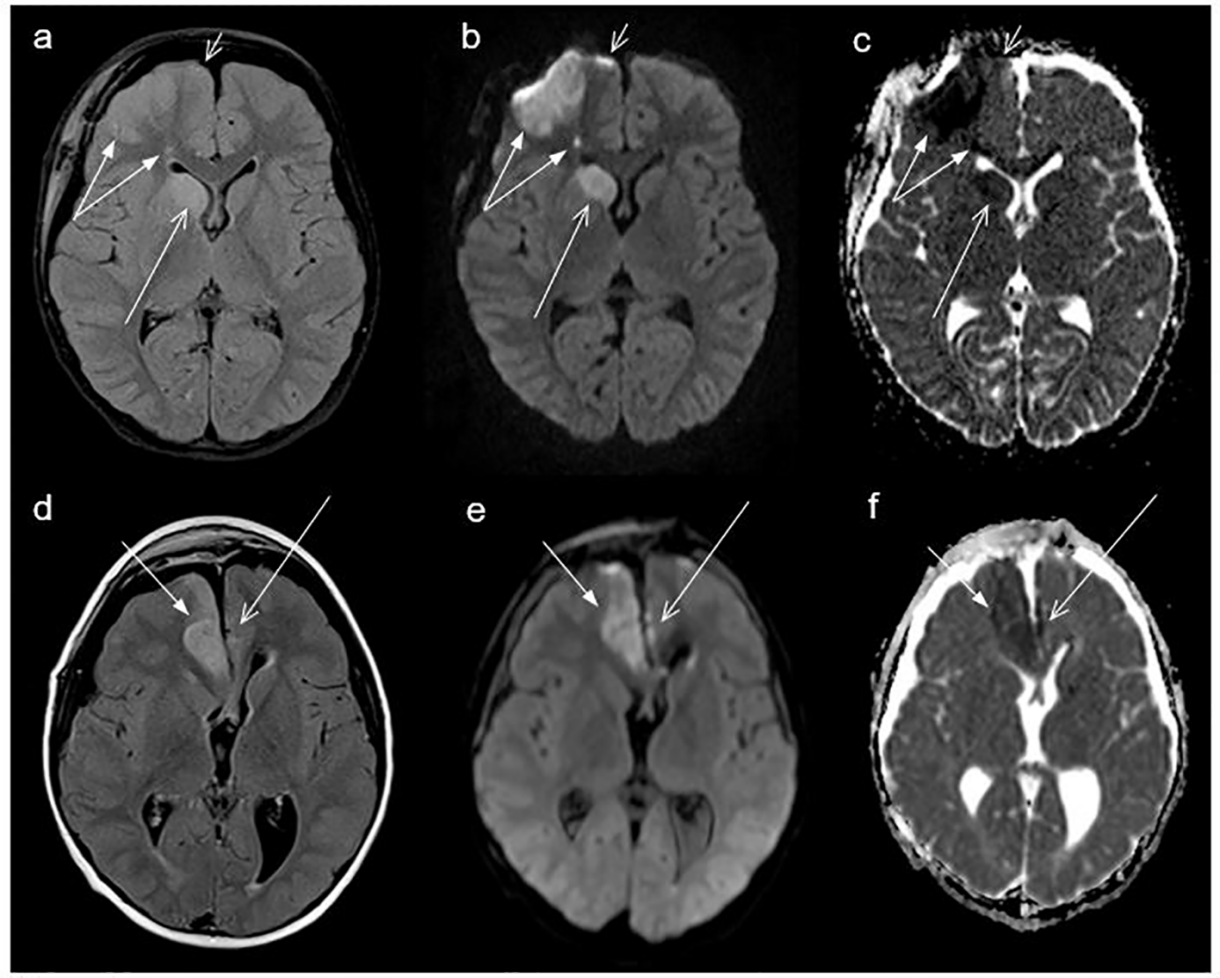
Cranial magnetic resonance imaging (MRI) of a craniopharyngioma patient (case #25, **Table 2**) with cerebral infarction (CI) of middle cerebral artery (MCA) on the right side (double arrow, territorial CI), anterior cerebral artery ACA (Heubner) right side (long arrow), and the top of ACA right side (short arrow, paired with contusions), performed 4 days after surgery **(A–C)**; and MRI of a craniopharyngioma patient (case #19, **Table 2**) with cerebral infarction (CI) of the anterior cerebral artery (ACA) on the right side (short arrow) and on the left side (long arrow) showing a linear cortical CI, MRI performed one day after surgery **(D–F)**. **(A, D)** show fluid-attenuated inversion recovery (FLAIR) sequences, **(B, E)** show DWI b1000, and **(C, F)** show apparent diffusion coefficient (ADC) images, all axial plane.

Pre- and postsurgical MRIs were reference-assessed by neuroradiologists (B.B. and D.G.) for the presence of CI. The imaging features of CI were defined as high MRI signal intensity on T2-weighted and/or proton density-weighted and/or fluid attenuation inversion recovery (FLAIR) weighted and/or restricted diffusion-weighted imaging (DWI) with high signal on the b1000 images and a low acquired diffusion coefficient (ADC) with the typical shape of an ischemic lesion (**Figure 1**). Preoperative imaging was also checked in patients with CI, for ischemic lesions to verify that CI was definitely associated to therapeutic interventions since DWI was not performed during all postoperative MRI examinations and some postoperative MRIs were too late to detect restricted diffusion. Furthermore, in CP patients with CI the vessel territory of the CI area, the maximum diameter of ischemic lesion on the first postoperative MRI and postoperative clinical complaints were analyzed.

### Clinical Parameters

In all CP patients, clinical and auxiological parameters were analyzed based on the protocol of the KRANIOPHARYNGEOM 2007 trial (15). Body weight, body height (SDS) (20), and body mass index (BMI) were measured at the time of CP diagnosis and prospectively at 3-months intervals after diagnosis. BMI (w/h^2^; w= weight/kilogram, h= height/meter) was calculated as standard deviation score (SDS) according to the age-related references of Rolland-Cachera et al. (21) for each subject at diagnosis of CP and at defined time points (one and 3 years after CP diagnosis, and at last follow-up visit).

### Quality of Life Questionnaires

The Pediatric Quality of Life (PEDQOL) (22, 23) questionnaire was used to assess health-related QOL in patients diagnosed with CP at an age ≥5 years, at defined time points (3, 12 and 36 months after diagnosis of CP). In CP patients younger than 18 years of age at the time of study, parental assessment of CP patient QOL was obtained using the PEDQOL questionnaire. The PEDQOL instrument is defining health-related QOL within the domains: autonomy, emotional stability, body image, cognition, physical function, social functionality in family, and among friends. A high PEDQOL score is equivalent to more negative self- or parental-assessed QOL (22).

To analyze functional capacity, we used the German daily life ability scale Fertigkeitenskala Münster-Heidelberg (FMH) at the defined time points 3 months, one and 3 years after diagnosis of CP and at last visit (24). Based on 56 items, the FMH instrument measures the capacity for routine, daily life actions. FMH was normalized with 971 individuals (45% female), aged between 0 and 102 years. FMH scores have the format of age-dependent percentiles (25). The time for answering the FMH questionnaire is in average 4.5 minutes (24).

### Statistical Methods

Statistical analyses were performed using SPSS 26 (SPSS, Inc.) and SAS software, version 9.4 for Windows, SAS Institute, Cary, NC, USA. Data are displayed as median (range) or frequency (percent). For comparison of continuous variables between two independent groups, the Mann-Whitney U test was used. In order to analyse possible risk factors for CI, logistic regression was used. The final model was chosen by a stepwise selection algorithm. Results are shown as odds ratio (OR) and corresponding 95%-confidence interval (CI). All inferential statistics are intended to be exploratory (hypotheses generating), not confrimatory, and are interpreted accordingly.

## Results

**Figure 2:**
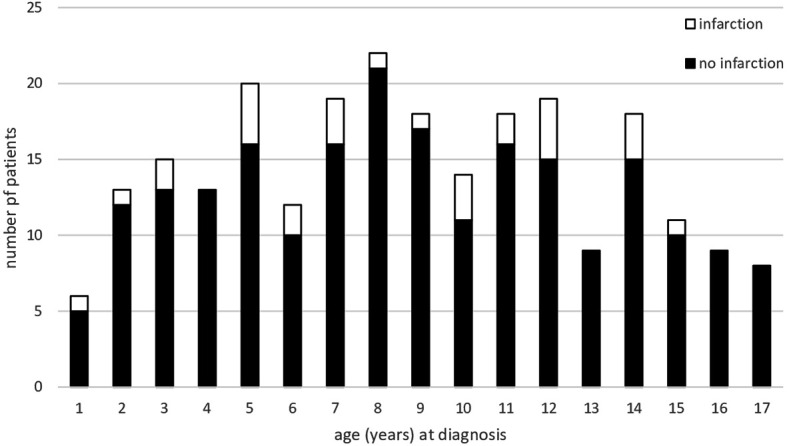
Age of 244 patients (recruited in KRANIOPHARYNGEOM 2007 between 2007 and 2019) at the time of primary surgery/diagnosis of adamantinomatous, childhood-onset craniopharyngioma. Patients presenting with cerebral infarction (CI) are represented by open parts of columns; patients without CI are depicted as solid black parts of columns.

Twenty-eight of 244 patients (11%) developed CI based on central review of MRI. Patients developing CI were comparable to patients without CI in terms of gender, age at CP diagnosis (**Figure 2**), BMI, height SDS, grade of HI and tumor location at the time of CP diagnosis. There was a trend (p=0.094) towards larger tumors in 28 patients with CI (median tumor size: 25.1 cm^3^; range: 0.01–187 cm^3^) when compared with patients without CI (median tumor size: 15.7 cm^3^; range: 0.01–286 cm^3^). A hydrocephalus was diagnosed in 22 of 28 patients with CI (79%), whereas only 76 of 216 patients without CI (35%) presented with hydrocephalus at the time of CP diagnosis (p<0.001) (**Table 1**).

**Table 1:** Characteristics of the study population showing data for patients with and without cerebral infarction (CI) recruited in KRANIOPHARYNGEOM 2007 between 2007 and 2019.

	Patients	Patients	*Case 1*	*Case 2*	*P value*
	without CI	with CI			
**Patient number, n**	216	26	1	1	
**Gender, female/male, n (%)**	113 (52)/103 (48)	11 (42)/15 (58)	female	male	0.423^a^
**Age at diagnosis (year)**	9.2 (1.3 – 17.9)	10.1 (1.7 – 15.6)	5.0	2.0	0.642^a^
**Duration of history (months)**	5 (0.1 – 108)	7 (0.1 – 96)	36.0	0.5	0.735^a^
**Follow-up time (year)**	4.9 (0.2 – 13.1)	3.7 (0.1 – 9.9)	5.8	2.0	0.080^a^
**BMI at dgx [SDS (**21**)]**	0.4 (-11.8 – 10.0)	1.5 (-3.0 – 9.1)	2.9	-1.3	0.058^a^
**Height at dgx [SDS (**20**)]**	-1.0 (-4.9 – 3.6)	-1.0 (-4.2 – 1.8)	-1.1	1.8	0.974^a^
**BMI at last visit [SDS (**21**)]**	3.0 (-2.0 – 20.8)	4.4 (-1.1 – 13.2)	4.1	0.7	0.044^a^
**Height at last visit [SDS (**20**)]**	-0.3 (-3.9 – 3.1)	0.1 (-4.1 – 2.5)	-0.1	1.7	0.216^a^
**Tumor volume (3D cm³)**	15.7 (0.01 – 286.3)	25.1 (0.01 – 187.6)	11.5	13.4	0.094^a^
**Hydrocephalus, n (%)**	76 (35.2)	21 (80.8)	x		<0.001^a^
**Frequency of surgical interventions, n (%)**	2 (1 – 12)	2 (1 – 7)	4	1	0.528^a^
**Degree of first surgical resection, n (%)**					0.091^b^
**Complete resection**	49 (23)	10 (39)			
**Incomplete resection**	167 (77)	16 (62)		x	
**no resection**	0	0	x		
**Surgical approach at first resection, n (%)**					0.578^b^
**Bifrontal**	17 (8)	2 (8)			
**Endoscopy**	4 (2)	1 (4)	x		
**Posterior fossa**	1 (0.5)	0			
**Transsphenoidal**	38 (18)	2 (8)			
**Transventricular/transcallosal**	8 (4)	1 (4)			
**Unilateral, fronto-temporal or variations of this approach**	145 (67)	20 (76)		x	
**n.a.**	3 (1)	0			
** Transsphenoidal *vs.* “other”**	38/175	2/24			0.269^b^
**Surgical experience (patient load/year)**					0.611^b^
**≤1 patient per year/centre, n**	174 (80)	20 (77)	x	x	
**>1 patient per year/centre, n**	42 (20)	6 (23)			
**Hypothalamic involvement (HI), n (%)**					0.219^b^
**No HI**	11 (5)	0			
**Anterior HI**	60 (28)	4 (15)		x	
**Anterior and posterior HI**	144 (67)	22 (85)	x		
**n.a.**	1 (1)	0			
**Hypothalamic lesion (HL), n (%)**					0.0007^b^
**No HL**	61 (28)	0		x	
**Anterior HL**	80 (37)	11 (42)			
**Anterior and posterior HL**	74 (34)	15 (58)	x		
**n.a.**	1 (1)	0			

BMI, body mass index; SDS, standard deviation score; pts., patients; yr., year; HI, presurgical hypothalamic involvement; HL, surgical hypothalamic lesion; n.a., data not available. ^a^all patients with CI, including case #1 and case #2, ^b^patients with CI, except case #1 and case #2. Depicted are medians and ranges in paraenthesis or frequency and percentage in parenthesis.

One CI (case #2, **Table 2**) was detected before initial surgery. At the time of diagnosis, MRI showed a suprasellar tumor with close connection and displacement of the right internal carotid artery as well as anterior and medial cerebral arteries. A large cystic tumor compartment showed typical MRI signs of an intracystic hemorrhage leading to CI. Another CI (case #1, **Table 2**) occurred during a surgical procedure replacing an intracystic catheter, which did not drain properly due to occlusion. All other 26 cases of CI (96%) were detected after initial tumor resection at a median interval between surgery and CI diagnosis of one day, ranging from 0.5 to 53 days. In one case of CI (case #21, **Table 2**), an additional left frontal lobe bleeding was observed. 12 of 28 patients with CI were irradiated (2 photon XRT; 10 proton beam therapy). In all irradiated cases, CI was diagnosed before the start of irradiation (XRT) (median interval between CI diagnosis and XRT: 13 months; range: 1.8 – 34.5 months).

**Table 2:** Characteristics of 26 patients with childhood-onset craniopharyngioma (CP) patients (recruited in KRANIOPHARYNGEOM 2007 between 2007 and 2019) with cerebral infarction (CI) confirmed by central neuroradiological review.

No.	Sex (f/m)	Age at surgery (yr)	CP-Volume at dgx (cm³)	HI (grade)	Degree of surgical resection	Surgical approach (1,2,3,4)	Intra.-operative vascular lesions	HL (grade)	Interval btw. surgery and CI detection (days)	Cerebral infarction (CI)	Volume of CI (cm³)	Cerebral infarction (CI)	Volume of CI (cm³)	Cerebral infarction (CI)	Volume of CI (cm³)	age at XRT (yr)
**1**	f	5.5	11.5	2	None	4	/	2	0	AChA ri.	3.2	/	/	/	/	7.0
2*	m	2.0	13.4	1	Incompl.	1 ri.	/	0	-3 before OP	MCA ri. multifocal	2.0	/	/	/	/	/
**3**	m	11.8	10.7	2	Compl.	1 ri.	/	2	2	ACA le. basal	13.5	ACA ri. basal	14.4	MCA ri. frontal	72	/
**4**	m	15.6	1.9	2	Compl.	1 ri.	/	2	53	ACA ri. (Heubner)	3.5	MCA ri. BG multifocal	0.7	MCA ri. temporal	1.6	/
**5**	f	8.0	187.6	2	Incompl.	2	/	2	0	ACA multifocal ri.+le.	0.9	AChA ri.	0.3	/	/	9.8
**6**	m	2.4	33.5	2	Incompl.	1 ri.	/	1	2	MCA ri. frontal	0.6	/	/	/	/	2.5
**7**	f	12.0	19.4	2	Compl.	1 ri.	/	2	18	ACA ri. (Heubner)	0.6	/	/	/	/	
**8**	m	3.6	28.4	2	Compl.	2	/	1	0	ACA ri. frontobasal	11.9	ACA le. frontobasal	6.5	/	/	5.5
**9**	f	10.4	26.2	2	Incompl.	1 ri.	Surgical vas-cular lesion	2	7	AChA ri.	1.8	/	/	/	/	/
**10**	m	14.5	15.4	2	Incompl.	1	/	2	11	ACA ri.	1.8	/	/	/	/	/
**11**	m	3.5	74.8	1	Compl.	1	/	1	4	ACA ri. (Heubner)	0.5	ACA le. (Heubner)	0.8	MCA le. BG	0.4	/
**12**	m	9.8	28.1	2	Incompl.	1 ri.	Arterial tumor bleeding	1	0	ACA ri.	2.8	ACA + ACM le.**	/	/	/	10.7
**13**	f	8.5	3.4	1	Compl.	1	/	1	1	ACA ri.	3.3	ACA le.	1.0	/	/	/
**14**	m	14.8	6.4	1	Incompl.	1 le.	/	1	1	ACA ri. basal	6.3	ACA le. basal	1.9	/	/	/
**15**	f	10.5	13.8	2	Incompl.	1	/	2	2	ACA ri. (Heubner)	9.8	/	/	/	/	/
**16**	m	6.4	36.6	2	Incompl.	1 ri.	/	2	0	MCA ri. cortical***		ACA ri. hochfrontal	0.03	/	/	7.4
**17**	m	10.5	30.8	2	Incompl.	1	Surgical vas-cular lesion	2	1	ACA li. basal	2.5	ACA ri. basal	0.6	/	/	11.5
**18**	m	9.2	13.7	1	Incompl.	1 ri.	/	1	1	MCA ri., capsula int.		/	/	/	/	/
**19**	m	6.0	31.9	2	Compl.	1 ri.	/	2	1	ACA ri. basal	10.4	ACA le. basal	1.6	/	/	/
**20**	f	12.5	10.7	2	Compl.	1 ri.	Surgical vas-cular lesion	2	0	MCA ri.	1.1	MCA ri.	0.5	/	/	15.4
**21**	f	7.2	14.1	2	Compl.	1	/	2	0	ACA le. basal	0.7	ACA ri. basal		/	/	/
**22**	m	11.7	57.6	2	Incompl.	1 le.	Surgical vas-cular lesion	2	1	ACA le. (Heubner)	6.0	PCA ri.	0.3	/	/	/
**23**	m	12.7	0.01	2	Incompl.	4	Surgical vas-cular lesion	2	1	ACA ri. (Heubner)	3.1	ACA le. (Heubner)	4.6	/	/	/
**24**	f	12.2	32.0	2	Incompl.	3	Venous bleeding	1	1	PCA le.	0.4	ACP ri.	0.3	PCA le.	1.7	/
**25**	f	5.4	17.9	2	Incompl.	1 ri.	/	2	4	MCA ri. ****		ACA ri. (Heubner)	3.2	ACA ri.	0.2	6.5
**26**	f	5.9	94.2	2	Compl.	1 le.	/	1	1	ACA ri. (Heubner)	9.0	ACA ri. frontobasal #	/	ACA le. basal #	/	7.7
**27**	m	7.7	107.2	2	Incompl.	1 ri.	/	1	1	ACA ri. (Heubner)	3.7	/	/	/	/	7.9
**28**	f	14.2	24.0	2	Incompl.	3	surgical vas-cular lesion	1	1	ACA ri. (Heubner)	0.06	/	/	/	/	15.3

ACA, anterior cerebral artery; MCA, middle cerebral artery; PCA, posterior cerebral artery; AChA, anterior choroidal arteryri., right; le., left; m, male; f, female; yr, year; ant., anterior; compl., complete; incompl., incomplete; int., interna; Surgical approach: 1=unilateral, 2=bifrontal, 3=transsphenoidal, 4=endoscopical; BG = Basal ganglia; * patient with CI detected before initial surgery; ** cortical along frontal lobe reaching the insula, precise measurement not possible, <25% ACA and MCA region; *** precise measurement not possible, < 75% of MCA region; **** cortical frontal with temporopolar extension, precise measurement not possible, 25-50% of MCA region; # (linear along the border of resection), precise measurement not possible.

In further analyses, we excluded the above-mentioned two cases (case #1 and case #2, **Table 2**) and analyzed the remaining 26 CP patients with initial surgical intervention leading to CI. The realized degree of surgical resection was different between 26 CI and 216 no-CI patients. Gross-total resection (GTR) was performed more frequently in CI patients (39%) when compared with non-CI patients (23%). With regard to surgical approaches, we observed that a transsphenoidal route was chosen in 38 of 216 patients without CI (18%) and in 2 of 26 patients with CI (8%). As a potential measure of surgical experience, we analyzed also data for CP patient load of neurosurgical centers and found no associations between patient load and rate of CI (**Table 1**).

Analyzing surgical reports with regard to intraoperative complications leading to CI, in 7 of 26 patients (27%) bleeding complications resulting in CI have been reported. In 14 of 26 patients no intraoperative complications resulting in vascular lesions with consecutive bleeding were mentioned in surgical reports. The outcome of 7 patients with intraoperative bleeding was comparable with the outcome in 20 CI patients without intraoperative bleeding episodes mentioned in surgical reports with regard to QOL and functional capacity (data not shown).

Whereas the rate of presurgical HI was similar in patients with and without CI, patients with CI were characterized by a different pattern of surgical HL when compared with patients without CI. Patients without CI more frequently showed no surgical HL (28% *vs.* 0%), whereas patients with CI had a higher rate of anterior and posterior hypothalamic surgical lesions (58% *vs.* 34%) (p=0.0007) (**Table 1**). In 46% of all CI cases, CI occurred in both cerebral hemispheres mainly after bifrontal surgical approach (38%). In 54% of CI, bleeding was observed in one hemisphere – right-sided in all cases, after right-sided surgical approach in 73% (**Table 2**).

Logistic regression analysis including age at diagnosis, initial tumor volume, hydrocephalus, degree of surgical resection, and surgical HL as potential risk factors for CI, showed that hydrocephalus and GTR were relevant risk factors for CI. Both were included in a multivariable logistic regression model. The risk of CI was increased [OR = 7.7, 95%-confidence interval (2.70, 21.72)] when patients initially presented with hydrocephalus or when GTR was achieved [OR = 2.76, 95%-confidence interval (1.09, 6.86)] (**Table 3**).

**Table 3:** Result of univariable logistic regression for potential risk factors of cerebral infarcts (CI) and multivariable logistic regression model for CI chosen by a stepwise selection algorithm in patients with childhood-onset craniopharyngioma recruited in KRANIOPHARYNGEOM 2007 between 2007 and 2019.

Effect	Comparison	p-value	Odds ratio
		(Wald)	(95% confidence interval)
	**Univariable logistic regression model for potential risk factors of CI**		
Age at diagnosis (n = 242)	per year	0.9637	0.998 (0.908; 1.096)
Sex (n = 242)	female *vs.* male	0.3372	0.668 (0.294; 1.522)
Initial tumor volume (n = 220)	per cm³	0.2753	1.005 (0.996; 1.013)
Grade of HI (n = 241)	2 *vs.* 1 or 0	0.0762	2.711 (0.900; 8.167)
Hydrocephalus (n = 222)	yes *vs.* no	0.0003	6.632 (2.399; 18.331)
Gross-total resection (n = 242)	yes *vs.* no	0.0819	2.130 (0.909; 4.993)
Grade of HL (n = 241)	2 *vs.* 1 or 0	0.0237	2.598 (1.136; 5.943)
	**Multivariable logistic regression model for CI chosen by a stepwise selection algorithm**		
Hydrocephalus	yes *vs.* no	0.0001	7.652 (2.695; 21.722)
Gross-total resection	yes *vs.* no	0.0319	2.757 (1.092; 6.958)

HI, preoperative hypothalamic involvement; HL surgical hypothalamic lesion.

**Figure 3:**
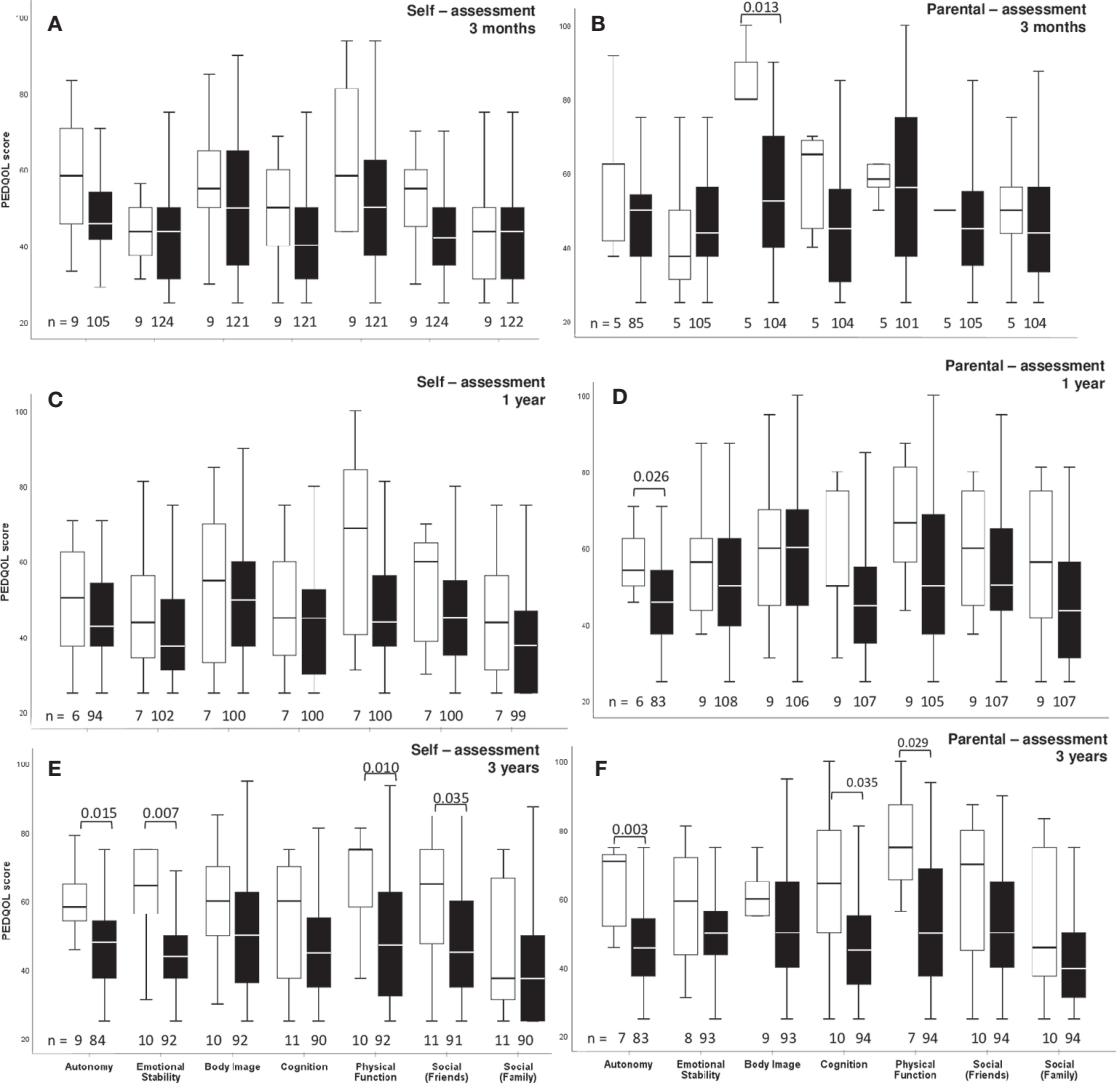
Self-assessment **(A, C, E)** and parental assessment **(B, D, F)** of quality of life by Pediatric Quality of Life questionnaire (PEDQOL) in childhood-onset craniopharyngioma (CP) patients, recruited in KRANIOPHARYNGEOM 2007 between 2007 and 2019, with regard to cerebral infarction (CI) confirmed by central neuroradiological review. White boxes: CI, and black boxes: no CI. PEDQOL scores are shown as negative rating at the time points three months **(A, B)**, one year **(C, D)**, and three years **(E, F)** after CP diagnosis. The horizontal line in the middle of the box depicts the median. The top and bottom edges of the box respectively mark the 25^th^ and 75^th^ percentiles. Whiskers indicate the range of values that fall within 1.5 box-lengths.

**Figure 4:**
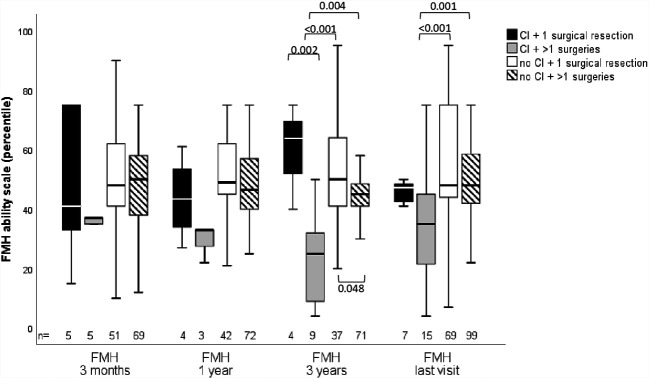
Functional capacity as measured by capability scale Fertigkeitenskala Münster Heidelberg (FMH) in childhood-onset craniopharyngioma (CP) patients (recruited in KRANIOPHARYNGEOM 2007 between 2007 and 2019) with or without cerebral infarction (CI) and with a single or multiple surgical interventions at the time points 3 months, one year, 3 years after CP diagnosis, and at the time of last visit.

QOL as measured by PEDQOL was lower for CI patients at the time points 3 months, one, and 3 years after CP diagnosis in self-assessment as well as parental assessment (**Figure 3**). Furthermore, functional capacity as measured by FMH was reduced one year after CP diagnosis (p=0.014), 3 years after CP diagnosis (p=0.024) and at last visit in patients with CI (p<0.001), when compared with CP patients without CI. When functional capacity was compared between CI and no CI patients with regard to the frequency of surgical interventions, we observed that FMH scores were reduced in CI patients with more than one surgical intervention (**Figure 4**).

## Discussion

Long-term outcome after CP is frequently impaired by morbidity due to hypothalamic obesity including cardiovascular (26) and neurovascular disease (27). Neurovascular complications such as CI in CP may result from injury to any of the major intracranial vessels and their branches, due to tumor location/infiltration and/or treatment-related lesions such as surgical injury, postoperative vasopasms or XRT-induced vessel damage.

Increased rates of cerebrovascular disease have been reported in a number of studies after pituitary XRT. In a series of 156 patients with sellar masses, increased CI rates were found in patients with higher administered XRT doses (28). Cerebral XRT was also reported to be associated with increased risk for Moyamoya syndrome. In patients treated with XRT for primary brain tumors, the estimated prevalence of Moyamoya syndrome ranges from 3.5% to 9% (29–31). Ullrich et al. (29) observed that, Moyamoya was diagnosed in 12 of 345 patients (3.5%), at a median follow-up of 54 months after XRT for brain tumors.

CI or ischemic stroke after XRT of a skull base tumor, is usually a delayed event (28, 32–36) and most of the patients in a study of Astradsson et al. (37) had a stroke with onset several years after XRT. Tumors of the anterior skull base are located close to anatomical structures such as the Circle of Willis and the cavernous sinus, so that collateral XRT of these structures may occur and lead to vascular sequelae (38, 39).

We have previously reported on fusiform dilatations of the internal carotid artery (FDCA) representing a potential complication after surgery for childhood-onset CP with suprasellar extension. We could show that FDCA is a extremely rare complication associated with surgical treatment of CP patients without relevant impact on prognosis and clinical outcome after CP (27).

In the literature, associations between CI and hypothalamic lesions, release of inflammatory substances during neurosurgical resection, and direct surgical injury to the blood vessels in the basal cisterns have been reported (40, 41). During and after CP surgery, vasospasm may occur due to spillage of CP cyst fluid inducing chemical meningitis (42). Spontaneous rupture of CP cysts inducing preoperative vasospasm has been observed (42). Arterial spasms of femoral vessels have been reported in animal models four days after instillation of cyst fluid on the femoral vessel (43). Diabetes insipidus and consequent hypovolemia need close peri and postoperative monitoring as potential risk factors for CI. Diabetes insipidus results in volume deficits. Accordingly, cerebral hypoperfusion and ischemia could be worsened in cases of intravascular fluid depletion.

Wijnen et al. (44) reported on an increased risk for CI after CP (SIR: 4.9, 95%-confidence interval: 3.1, 8.0). The excess risk for CI was higher in female patients with childhood-onset CP, and in patients with hydrocephalus and CP recurrence.

In our cohort of the German Craniopharyngioma Registry, 11% of all CP cases developed CI. In one patient, CI occurred before surgical and radiooncological treatment, indicating that the tumor disease itself represents a certain risk for CI. In 27 of 28 cases, CI occurred following surgical procedures. Intraoperative bleeding was mentioned in the records of only 25% of cases with CI, indicating that pathogenic mechanisms different from surgical vascular damage could also play a role in CI. The risk for CI was not gender or age-related in our study. We observed an association between CI and increased intracranial pressure and the degree of surgical resection. We speculate that sudden shifts in intracranial pressure and brain shifts due to hydrocephalus treatment and tumor resection results in circulatory changes. Such changes – especially when associated with hypovolemic episodes due to diabetes insipidus - are hypothesized to increase the risk for CI. Accordingly, a two-stage surgical treatment strategy could be discussed in CP patients presenting with initial hydrocephalus: 1.) decreasing and stabilizing intracranial pressure by drainage of cerebrospinal fluid (CSF); 2.) stabilizing salt and fluid homeostasis by sufficient desmopressin substitution, and 3.) definite surgical intervention for tumor resection without vascular or hypothalamic damage after a time of sufficient stabilization achieved for 1.) and 2.). However, as CI in one of our (cases #1) occurred after cyst drainage, it is important to be aware that rapid changes in intracranial pressure – as caused for instance by cyst drainage or presumably also by CSF drainage – could be a potential risk factor on its own for CI even without a concomitant resecting intervention. Further studies on prevention of CI are warranted to answer controversies on this speculation. Interestingly, XRT was not associated with increased risk for CI. However, longer follow-up is necessary to estimate long-term risks of XRT for vascular events such as CI.

In multivariable analysis, radical surgical strategies such as GTR had independent impact on the risk for CI. Patients with CI had a higher rate of GTR when compared with CP patients without CI. We recommend limited surgical strategies in order to preserve hypothalamic integrity and functionality for prevention of special risks for CI and deterioration of long-term QOLand functional capacity (2). Reduced functional capacity was also associated with high frequency of surgical interventions after CI diagnosis.

Due to retrospective analysis and small cohort size, the results of our study are limited. At this point, some of our observations and conclusions are speculative. However, given the high degree of completeness with regard to multicentre recruitment of patients with childhood-onset CP in KRANIOPHARYNGEOM 2007, our study has the advantage to provide reliable data on the rate of CI in these patients. The exact time point of each CI cannot be confirmed based on our data. Diagnosis of CI is based on centrally reviewed MRI, which has been performed postoperatively and at 3-months intervals after primary diagnosis as part of the KRANIOPHARYNGEOM 2007 study protocol. The data on intraperative surgical vascular lesions are based on the available surgical records.

We conclude that especially in CP patients with initial hydrocephalus and surgical procedures such as GTR leading to complete resection in complicated tumor locations/adhesion of vessels, the risk of CI is increased. CI leads to severe impairment of QOL and functional capacity during long-term follow-up after childhood-onset CP.

## Data Availability Statement

The raw data supporting the conclusions of this article will be made available by the authors, without undue reservation.

## Ethics Statement

The study KRANIOPHARYNGEOM 2007 (Clinical trial registration number: NCT01272622) was approved by the local standing-committee on ethical practice of the Medizinische Fakultät, Julius-Maximilians-Universität Würzburg, Germany (140/99; 94/06, respectively). Written informed consent to participate in this study was provided by the participants’ legal guardian/next of kin.

## Author Contributions

SB researched the data and wrote and reviewed the manuscript. BB and DG did neuroradiological assessment of all imaging. BB is the neuroradiologist, who performs reference-assessment of imaging in all patients recruited in KRANIOPHARYNGEOM 2007. They prepared the imaging data and their presentation and reviewed/edited the manuscript. ME supervised statistical analyses and reviewed/edited the manuscript. CF and AO contributed to the analytical plan, data analysis/presentation and discussion and reviewed/edited the manuscript. As a neurosurgeon, JF contributed to the analytical plan and discussion and reviewed/edited the manuscript. HM initiated and conducted the multicenter trials HIT-Endo and KRANIOPHARYNGEOM 2000/2007, contributed to the analytical plan and discussion and reviewed/edited the manuscript. All authors contributed to the article and approved the submitted version.

## Funding

This study was funded by a grant (HM, DKS2014.13; BB, DKS2018.02) of the German Childhood Cancer Foundation, Bonn, Germany.

## Conflict of Interest

HM has received reimbursement of participation fees for scientific meetings and continuing medical education events from the following companies: Ferring, Lilly, Pfizer, Sandoz/Hexal, Novo Nordisk, Ipsen, and Merck Serono. He has received reimbursement of travel expenses from Ipsen and lecture honoraria from Pfizer.

The remaining authors declare that the research was conducted in the absence of any commercial or financial relationships that could be construed as a potential conflict of interest.
